# An integrated method for railway fastener defect detection and geometric parameter measurement using 3D line laser sensor

**DOI:** 10.1371/journal.pone.0341210

**Published:** 2026-05-22

**Authors:** Xiaocui Yuan, Wenyu Liu, Yongli Ma, Yongtao Wang, Baoling Liu

**Affiliations:** 1 School of Electrical Engineering, Nanchang Institute of Technology, Nanchang, China; 2 College of Control Science and Engineering, Zhejiang University, Hangzhou, China; 3 Jiangxi Gandian Electric Co., Ltd., Fuzhou, China; University of Vigo, SPAIN

## Abstract

Railway fasteners are key components that maintain track stability and ensure train operation safety. Automatic detection technologies for fastener defects have been widely adopted, but high-precision measurement of fastener geometric parameters still relies on manual operation, which is associated with low efficiency and significant measurement errors. This paper proposes an integrated method for railway fastener defect detection and geometric parameter measurement based on a 3D line laser sensor. A 3D imaging system is constructed to acquire RGB depth images and corresponding point clouds with a precise one-to-one mapping relationship. The YOLOv8 network is used to detect visual defects and locate intact fastener regions from RGB depth images, which are then mapped to the point clouds to extract fastener point cloud data. The PointNet++ network is adopted to segment fastener components, and the specifications of insulated blocks, the thicknesses of height adjustment pads, and bolt heights are calculated based on the spatial structure of fastener components. Experimental results demonstrate that the YOLOv8 model achieves 97.7% precision and 95.9% recall for visual defect detection, and 99.6% precision and 99.8% recall for intact fastener localization. All critical geometric measurements satisfy the corresponding railway engineering tolerances. 98.7% of HPuR measurements have errors below the 0.5 mm tolerance. HPuIP components with a 10 mm specification step exhibit a maximum measurement error of less than 2.5 mm, well below the 5 mm tolerance. 99% of insulated block measurements achieve errors below 0.5 mm tolerance at horizontal sampling intervals no greater than 0.4 mm. Bolt height measurement achieves no less than 90% precision and 91% recall for severe fastener loosening detection. The system operates at 4.32 km/h, making it suitable for hand-pushed on-site inspection. The proposed method realizes automatic defect detection and high-precision geometric parameter measurement in a unified framework, which can effectively replace manual inspection and significantly improve the intelligence and efficiency of railway track maintenance.

## 1. Introduction

**Fig 1:**
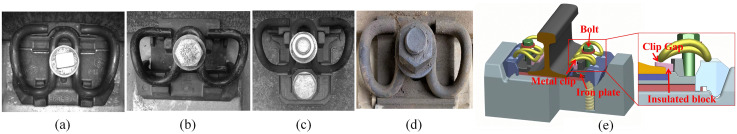
Common type of fastener on China’s railway and fastener structure. **(a)** Vossloh-300. **(b)** WJ-8. **(c)** WJ-7. **(d)** WJ-2; **(e)** fastener structure.

By the end of 2025, the operating mileage of China’s high-speed railways had exceeded 50,000 kilometers, accounting for more than 70% of the world’s total high-speed railway mileage [1]. Railway fasteners serve as critical infrastructure components that secure rails to sleepers, prevent rail misalignment, and guarantee the safety of train operations. According to China’s railway track design criteria, the mainstream fasteners for high-speed railways are Vossloh-300, WJ-8, and WJ-7 types (shown in Fig 1) which are primarily composed of track bolts, ω-shaped metal clips, insulated blocks, height adjustment pads, and other supporting components [2].

**Fig 2:**
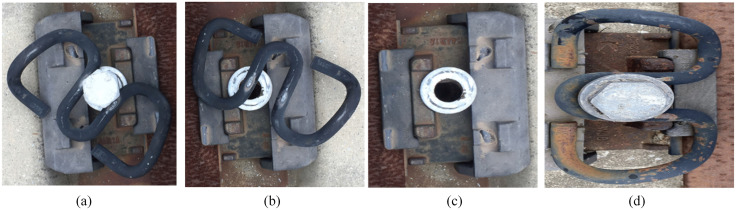
Common defects of fastener. **(a)** Skewed clip. **(b)** Bolt missing; **(c)** Fastener missing.; **(d)** Over-tight or looseness fastener.

During long-term service, the rail fastening system is susceptible to clamping force anomalies, which primarily manifest as either insufficient or excessive clamping force between the track bolt and the ω-shaped metal clip. Insufficient clamping force can result in bolt loosening, component displacement, and ultimately the detachment or loss of the metal clip, while excessive clamping force tends to induce fatigue fracture of the metal clip. Consequently, the common defects of railway fastening systems mainly include visual defects caused by positional deviation or deformation of metal clips, and structural defects caused by abnormal preload of fastening bolts, as shown in (Fig 2). These defects directly affect track regularity and ride comfort, and can even cause train derailment in severe cases, posing significant safety risks to railway operations.

Beyond functional defects, accurate measurement of fastener geometric parameters is equally critical for maintenance operations. The geometric parameters of fastener components primarily include the dimensional specifications of insulated blocks and height adjustment pads. During track fine-tuning for railway maintenance, insulated blocks and height adjustment pads need to be replaced with components of different specifications according to track geometry adjustment requirements, which imposes stringent demands on the precise measurement of fastener geometric parameters [3–5].

Currently, automatic fastener defect detection based on track inspection vehicles has been widely implemented, but the measurement of fastener geometric parameters still relies predominantly on manual operation, which suffers from low efficiency, high labor intensity, and susceptibility to measurement errors. Consequently, there is an urgent need to develop an integrated, automated methodology that simultaneously achieves high-precision fastener defect detection and geometric parameter measurement.

This paper proposes an integrated method for simultaneous railway fastener defect detection and geometric parameter measurement based on a 3D line laser measurement system. The remainder of this paper is organized as follows. The Related Work section reviews existing studies and identifies the key research gaps. The Methodology section presents the proposed method in detail. The Experimental Results and Analysis section verifies the performance of the proposed method through systematic experiments. The Discussion, Limitations and Future Work section discusses the limitations and application prospects of the method. Finally, the Conclusion section summarizes the core conclusions of this research.

## 2. Related work

Significant advances have been made in railway fastener inspection, particularly in defect identification. Existing mainstream solutions are generally categorized into two paradigms: 2D vision-based techniques and 3D vision-based techniques. However, research focused on high-precision geometric parameter measurements of fasteners remains notably limited in the literature.

### 2.1. 2D Vision-based fastener inspection methods

2D vision-based methods are the most widely adopted in industrial applications due to their low cost and easy deployment. This technology has been widely used for railway fastener defect detection, typically utilizing high-speed cameras to capture track grayscale images and processing these images to identify defective fasteners. Early studies relied on traditional image processing and machine learning methods, achieving fastener localization and defect classification through handcrafted feature extraction and classifier design [6–10]. However, these methods are highly dependent on specific application scenarios and lack sufficient robustness against complex environmental disturbances such as illumination variations and partial occlusion.

In recent years, deep convolutional neural networks (CNNs) have significantly enhanced the accuracy and robustness of visual defect detection for fasteners. The Faster R-CNN series, YOLO series, and CNN-Transformer hybrid networks have emerged as mainstream technical solutions [11]. For instance, Xiao et al. [12] Proposed an improved Faster R-CNN network to address the detection challenge of three critical types of small missing fastener components. Liu et al. [13] applied a modified Cascade Faster R-CNN framework for fastener defect detection. Nevertheless, inadequate feature extraction within backbone networks has constrained the performance of these models in complex real-world scenarios. To address this limitation, researchers have extensively explored various backbone enhancements and lightweight architectures, among which YOLO-series networks have become dominant due to their superior balance of speed and accuracy. For example, Hu et al. [14] enhanced the YOLOX-Nano framework by integrating adaptive spatial feature fusion and channel attention mechanisms. Qiu et al. [15]and An et al. [16] proposed improved YOLO frameworks, namely YOLO-FAM and T-YOLO respectively, for fastener defect detection.

However, data-driven deep learning models rely heavily on large-scale labeled defective samples for network training. To address the scarcity of defective samples, Liu et al. [17]employed a cycle-consistent adversarial network to generate ω-shaped defective fastener samples. They validated their approach by training and evaluating the model on combined datasets of generated and real defective images, achieving promising detection performance. Su et al. [18]mitigated the challenge of sample imbalance by leveraging geometric constraints and image inpainting to generate defective fastener samples, thereby enhancing defect detection accuracy. While these data augmentation methods effectively alleviate the problem of defective sample scarcity, they still struggle to simulate the extreme imaging conditions encountered in real-world railway environments. Specifically, severe rust accumulation and oil contamination on fastener surfaces often result in low-contrast, dark images, leading to high missed detection rates. To enhance detection accuracy in complex environments, He et al. [19] proposed a multi-scale CNN-Transformer hybrid network for railway fastener defect identification. Their experimental results focused exclusively on defective fastener detection performance and verified that their method outperformed mainstream state-of-the-art detectors, including the YOLO series and ViT-based object detection models. Despite the favorable performance of 2D vision-based methods in identifying visible surface defects, their fundamental limitation lies in the inability to capture spatial dimensional information, which restricts their capability in detecting structural defects and measuring geometric parameters.

### 2.2. 3D Vision-based fastener inspection methods

With the decreasing cost of 3D sensors, 3D vision-based methods have emerged as a promising solution to overcome the limitations of 2D methods. While 2D vision methods excel at detecting visible defects, they fundamentally lack the capability to assess structural integrity and measure precise dimensions. To address the critical challenge of detecting structural defects in fasteners with no explicit surface appearance anomalies, 3D vision technology, with its high-precision spatial topography perception capability, can effectively compensate for the inherent limitations of 2D vision in spatial information acquisition.

Mao et al. [20] pioneered a method for identifying fastener loosening defects by quantifying the gap between metal clip ends. They acquired high-density point cloud data using an integrated 3D structured light scanner, achieved accurate segmentation of metal clip point clouds via clustering algorithms, extracted the 3D skeleton of the point cloud to quantify the clip gap, and determined fastener tightness based on the measured gap value. Building on this foundational work, researchers have explored a variety of 3D imaging modalities for fastener inspection, including structured light scanning, fringe projection profilometry, and monocular depth estimation, for acquiring and quantitatively analyzing rail and fastener point clouds or depth images [21–24]. Shi et al. developed a 3D imaging system based on fringe projection profilometry, achieving fastener loosening defect detection by reconstructing fastener depth information in combination with measured bolt height differences [25]. Wang et al. [26] estimated the vertical depth values of bolts using the ZoeDepth model, achieved accurate segmentation of fastener components in conjunction with YOLOv8, and identified excessive loosening defects through quantified bolt height differences. These 3D vision-based techniques enable the detection of structural defects without obvious surface appearance changes, thus significantly improving the efficiency and reliability of fastener inspection.

Although modern visual inspection systems can simultaneously capture comprehensive data of all fastener components (bolts, clips, insulated blocks, height adjustment pads) as well as adjacent rails and sleepers during track scanning, existing studies have only exploited a small fraction of this data. Specifically, 2D methods rely solely on texture information for surface defect detection, while 3D methods only use depth data from bolts and clips for tightness assessment. However, research on the critical geometric parameters of insulated blocks and height adjustment pads has received limited attention in the existing literature. In automatic track fastener inspection, it is essential not only to detect defective fasteners but also to obtain dimensional and specification information for each fastener component. This enables dynamic establishment and updating of fastener archive information (including insulated block specifications, height adjustment pad thickness, etc.), which is critical for track regularity and gauge adjustment during intelligent railway operation and maintenance. Track fine-tuning is realized by adjusting the geometric parameters of the fastener system, yet parameter measurements of fasteners still rely on manual operation at present. Real-time measurement of geometric parameters for each fastener component while synchronously detecting fastener condition represents an effective approach to significantly improve railway track maintenance efficiency.

Therefore, this paper proposes an integrated method for simultaneous fastener defect detection and geometric parameter measurement based on a 3D line laser sensor. The main contributions of this research are summarized as follows:

(1) A railway track 3D measurement system is constructed using a 3D line laser sensor, which simultaneously acquires track RGB-depth images and their one-to-one registered point clouds, forming RGB-point cloud (RGB-P for short) bimodal data. This design leverages the rich texture information of RGB depth images for accurate visual defect detection and the precise spatial information of point clouds for high-precision geometric parameter measurement, enabling simultaneous completion of both tasks in a single scan.
(2) Accurate quantification of key geometric parameters of fasteners, including insulated block specifications and height adjustment pad thickness, is achieved with a measurement error below 0.5 mm for components with minimum specification differences of 1 mm.
(3) An integrated 3D measurement system for railway fasteners is developed, achieving real-time detection and measurement at a speed of 4.32 km/h and effectively replacing manual inspection operations.

## 3. Methodology

**Fig 3:**
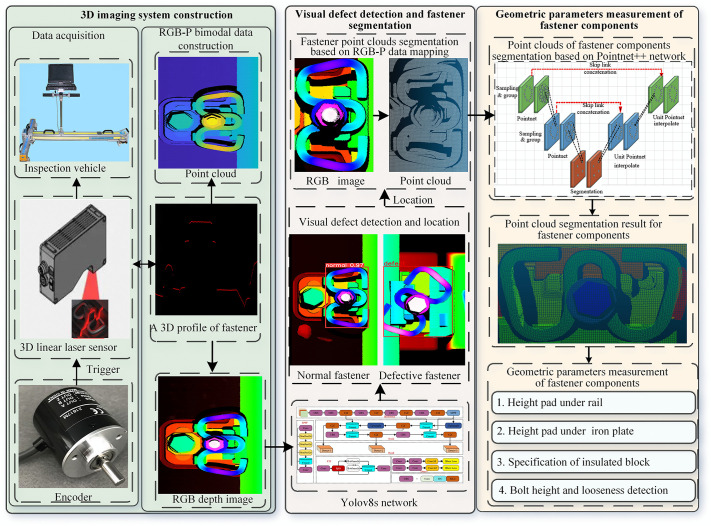
Flowchart of our method.

The workflow of the proposed integrated method is illustrated in (Fig 3), which consists of three core steps: (1) Construction of a 3D imaging system and acquisition of RGB-P bimodal data using a 3D line laser sensor; (2) Visual defect detection and fastener point cloud segmentation based on YOLOv8; (3) Fastener component segmentation using PointNet++ and geometric parameter measurement.

### 3.1. The 3D imaging system construction and RGB-P bimodal data acquisition

This section first describes the construction of the 3D line laser imaging system and the generation of RGB-P bimodal data. Then, it details the one-to-one mapping relationship between RGB depth images and point clouds, which forms the foundation for subsequent defect detection and geometric measurement.

#### 3.1.1. The 3D imaging system construction with 3D line laser sensor.

The Gocator 2450 3D line laser sensor is employed to collect railway track profile data. We vertically mount the sensor on a hand-pushed track inspection vehicle. The sensor operates based on the blue laser triangulation ranging principle, providing a Z-direction measurement accuracy of 0.01 mm and a maximum acquisition frequency of 5000 Hz. This performance specification meets the requirements for high-precision data acquisition in railway fastener inspection.

**Fig 4:**
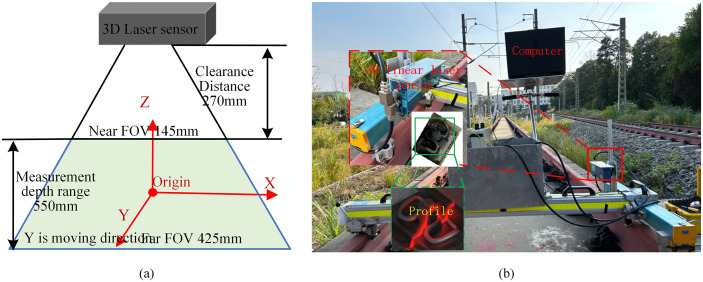
Principle and physical configuration of railway 3D imaging system based on 3D line laser sensor. **(a)** Field of view schematic of 3D line laser sensor; **(b)** Physical configuration of railway fastener 3D imaging system.

The sensor operates in external trigger mode, with trigger pulses provided by an encoder installed on the wheel of the inspection vehicle. For each trigger pulse, the sensor captures a single line of 3D profile data. A complete 3D point cloud of the track is generated through continuous scanning during the movement of the inspection vehicle. The 3D scanning system configuration is illustrated in (Fig 4). The scanning speed is synchronized with the traveling speed of the inspection vehicle (approximately 4 ~ 5 km/h), which satisfies the operational requirements of on-site manual inspection procedures.

#### 3.1.2. RGB-P bimodal data construction and acquisition.

The RGB-P bimodal data construction is designed to establish a precise one-to-one spatial correspondence between RGB depth images and point cloud data. This decoupled design intentionally avoids direct cross-modal feature fusion, which would introduce unnecessary computational complexity and potential information degradation due to modality mismatch. Instead, RGB depth images are used for fast visual defect detection, while point clouds are reserved for high-precision geometric measurements. This approach achieves both real-time detection efficiency and submillimeter-level measurement accuracy without compromising either capability.

For the scanned track area, the sampling intervals in the X-direction (perpendicular to the rail) and Y-direction (along the rail) are set as dx and dy, respectively. The number of points in each profile is calculated as N=W/dx, where W represents the measurement width in the X-direction. Similarly, M=L/dy profiles are acquired along the track direction (length L), forming a complete fastener point cloud. The sensor’s measurement depth range H is divided into Num segments with interval dh, with each segment assigned a distinct color representation.

**Fig 5:**
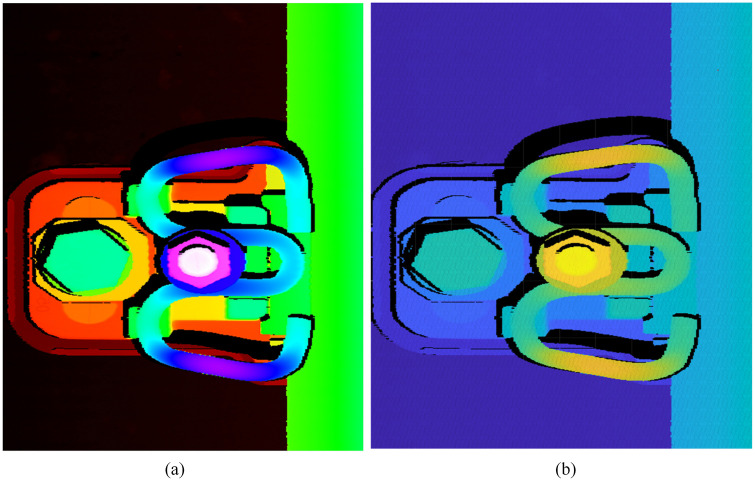
RGB-point cloud bimodal data of railway track. **(a)** RGB depth image showing fastener shape and presence; **(b)** Point cloud rendering showing spatial configuration and depth details. Black pixels in (a) represent invalid points due to sensor occlusions.

An empty image of size N×M is initialized to represent the texture of the corresponding point cloud. Each height value from the point cloud is mapped to the RGB depth image in coordinate order, generating a pseudo-color depth image where pixel values encode the corresponding point’s Z-coordinate that corresponds precisely to the point cloud data. As demonstrated in (Fig 5), pixels in the RGB depth image maintain a strict one-to-one correspondence with points in the point cloud. The RGB depth image effectively displays the fastener’s shape and presence, while the point cloud provides detailed spatial structure and depth information. This bimodal data representation enables visual defect detection from the RGB depth image and geometric parameter measurement from the point cloud data.

#### 3.1.3. The mapping relationship of RGB-P bimodal data.

In this research, the collected railway profiles are consolidated into a point cloud and recorded in a text file. Each line within this file contains three numerical values that represent *X*, *Y*, and *Z* coordinates of a single point. Given that the size of the RGB depth image is N×M, the text file contains N×M lines, each representing a point. The point clouds are then imported from the text file into a container of point structures, referred to as Vect.The number of points in the Vect container corresponds to the pixel count of the RGB depth image, with each point storing the X, Y, and Z coordinates of the point cloud. Given image I and its coordinates (i,j) the mapping relationship between image *I* and point cloud $P_{id}\left(X_{i},Y_{j},Z_{ij}\right)$ in Vect is described by Equation (1):


$$\begin{array}{c} \begin{array}{c} X_{i}=𝐕𝐞𝐜𝐭[i\times N+j].x\mathrm{\backslash hfill}\\ Y_{j}=𝐕𝐞𝐜𝐭[i\times N+j].y\mathrm{\backslash hfill}\\ Z_{ij}=𝐕𝐞𝐜𝐭[i\times N+j].z\mathrm{\backslash hfill} \end{array} \end{array}$$ (1)

Based on this established mapping relationship (Equation (1)), each pixel in the RGB depth image can be uniquely mapped to its corresponding point in the 3D point cloud. By leveraging the rich texture and color features of the RGB depth image, the fastener region can be rapidly segmented using image processing techniques. Subsequently, utilizing the precise one-to-one mapping relationship between the RGB depth image and the 3D point cloud, the corresponding point cloud data for the fastener region can be efficiently extracted.

### 3.2. Fastener defect detection, localization and point cloud extraction

Visual defects of fasteners manifest as conspicuous texture deviations from their normal states, whereas structural defects primarily manifest as height disparities. The line laser sensor operates on the triangulation principle, and the resulting RGB depth image is inherently unaffected by illumination variations, which significantly enhances the robustness of detection in complex environments. Additionally, the ω-shaped fastener exhibits a relatively simple geometric structure. For normal fasteners of the same type, the texture patterns in their RGB depth images are highly consistent. In contrast, fasteners with visual defects exhibit substantial shape and texture differences from their normal counterparts. Consequently, conventional deep learning-based object detection algorithms can efficiently identify fastener visual defects from RGB depth images.

Common deep learning object detection frameworks include the Fast-CNN series [27], the DETR series [28,29], and the YOLO series [30], among others. These frameworks have been extensively applied across diverse domains, each offering distinct advantages in terms of accuracy, speed, and computational requirements. In this study, the YOLOv8 network [31] is employed to detect visual defects of fasteners from RGB depth images. The use of rectangular bounding boxes enables rapid localization of fastener regions. By leveraging the bounding box coordinates and the established spatial mapping relationship between images and point clouds, the point cloud data corresponding to visually normal fastener regions can be efficiently segmented.

Considering the diverse nature of fastener visual defects and the similar appearance between normal fasteners and those with purely structural defects (which do not affect visual appearance), the proposed approach categorizes samples into two classes: “defect” for samples with obvious surface anomalies, and “normal” for both truly normal fasteners and structurally defective fasteners that maintain normal visual appearance. The trained YOLOv8 model generates a bounding box for each detection result, represented by (x,y,w,h), where (x,y) denotes the top-left corner coordinates of the rectangle, and w and h represent the width and height of the bounding box, respectively.

**Fig 6:**
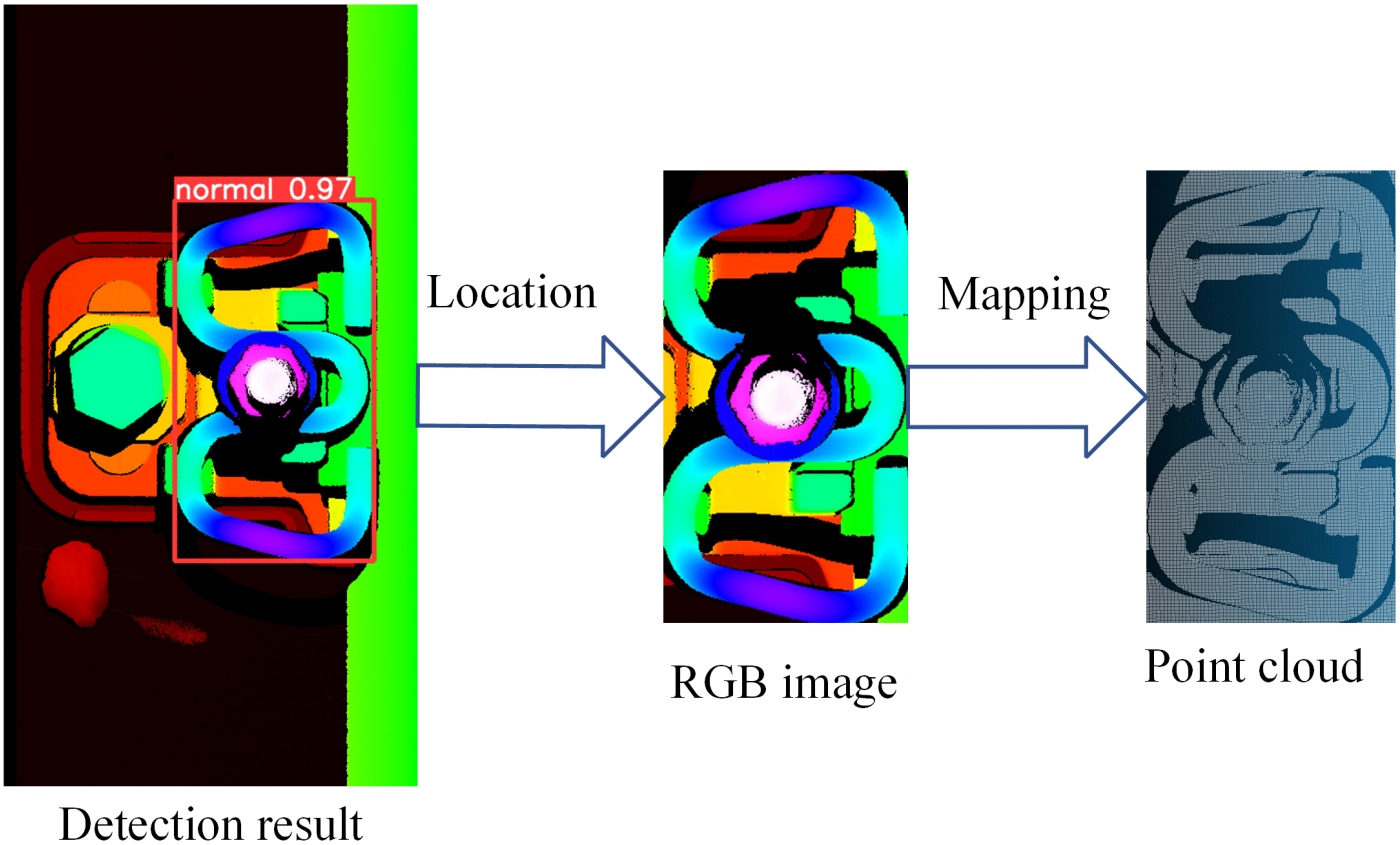
Flowchart of fastener point cloud segmentation based on RGB-P bimodal data mapping.

The bounding box coordinates (x,y,w,h) are utilized to precisely locate the visually normal fastener regions within the railway RGB depth images. Each pixel within the rectangular bounding box can be mapped to its corresponding 3D point in the railway point cloud using the spatial mapping relationship defined in Equation (1). This enables rapid and accurate segmentation of the fastener region’s point cloud from the complete track point cloud. The process and results of this concurrent segmentation are illustrated in (Fig 6). Specifically, the fastener region in the RGB depth image is efficiently mapped to the corresponding 3D point cloud region. This allows for fastener point cloud extraction simultaneously with visual defect detection, significantly enhancing computational efficiency for subsequent geometric parameter measurement tasks.

### 3.3. Intra-fastener component segmentation and geometric parameter measurement

Track maintenance operations frequently require precise adjustments of critical parameters, particularly the vertical alignment and gauge spacing between parallel rails. These precise geometric adjustments are fundamental to ensure safe and comfortable high-speed rail operations. During the operational service, track geometry gradually deteriorates due to dynamic loading and environmental factors, it is necessary for periodic adjustments to maintain optimal gauge and vertical alignment parameters. Long-term operations lead to variations in fastening system component geometries. Accurate measurement of these parameters is crucial for maintenance planning and component selection during track adjustment procedures. Traditional manual measurement methods are both time-consuming and prone to human error.

Precise measurement of fastener geometric parameters requires segmentation of fastener point clouds into individual components. The geometric parameters of these components can be computed by analyzing their spatial interrelationships and relative positions. Thus, this paper accurately segments the fastener point clouds to obtain the point clouds of each component of the fastener. The key geometric parameters of the fastener are then calculated based on the spatial relationships between these components.

#### 3.3.1. Fastener component segmentation based on PointNet++.

Point cloud segmentation methods can be broadly categorized into traditional approaches and deep learning-based methods [32]. Traditional methods (including region growing, K-means clustering, and RANSAC plane segmentation) exhibit inherent parameter sensitivity and poor adaptability to non-planar complex structures, making them unsuitable for high-precision fastener component segmentation. Among deep learning-based methods, PointNet++ [33] achieves an optimal balance between segmentation accuracy and computational efficiency by introducing hierarchical multi-scale feature learning. Although Point Transformer [34] delivers higher segmentation accuracy, its inference speed is approximately five times slower than that of PointNet++, which fails to meet the real-time requirements of railway inspection [34]. Therefore, PointNet++ is selected in this work for fastener component point cloud segmentation.

**Fig 7:**
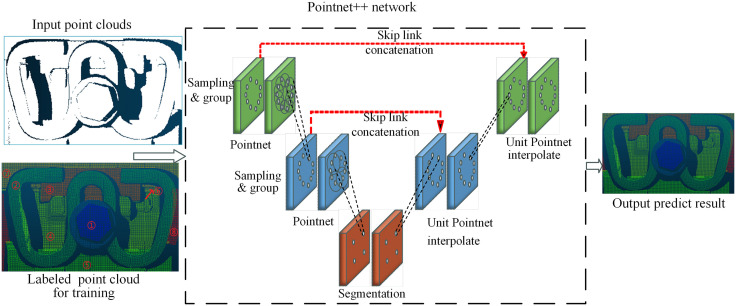
The architecture of pointnet++ network and schematic diagram of point cloud segmentation. Segmentation labels (①-⑧): ①: Upper surface of bolt; ②: Metal clip; ③: Insulated block; ④: Upper surface of iron plate; ⑤: Upper surface of gauge block; ⑥: Upper surface of rubber pad; ⑦: Rail edge; ⑧: Upper surface of sleeper.

PointNet++, proposed by Qi et al. [33], represents a milestone architecture for 3D point cloud understanding. As illustrated in (Fig 7), the network processes raw point clouds through a hierarchical feature learning encoder, followed by a task-specific prediction head. The hierarchical feature learning pipeline consists of two core operations: (1) sampling and grouping, and (2) local feature extraction using PointNet layers.

The sampling and grouping module uses farthest point sampling to select representative key points that achieve full coverage of the input space. To mitigate the influence of point cloud sparsity and non-uniform density, multi-scale grouping or multi-resolution grouping constructs spherical neighborhoods with varying radii around each sampled point, thereby effectively handling regions with different point densities. In the feature extraction stage, PointNet modules are applied to each local group to capture discriminative local features while preserving geometric topological relationships. This hierarchical feature aggregation mechanism encodes both point-wise characteristics and contextual information within local neighborhoods, enabling robust representation of complex geometric structures.

**Fig 8:**
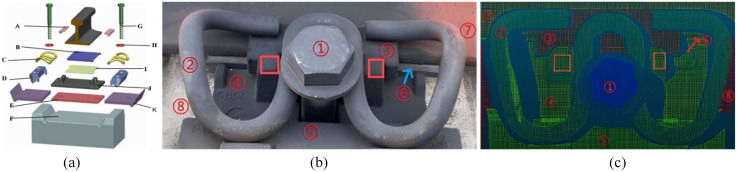
Geometric parameters measurement of WJ-8 fastener. **(a)** Schematic diagram showing key components; **(b)** In-situ image of the fastener; **(c)** Point cloud representation. Component labels **(A-K)**: A: Insulated block; B: Rubber pad; C: Metal clip; D: Gauge block; E: Buffer pad; F: Embedded brush; G: Bolt; H: Flat washer; I: HPuR; J: Iron plate; K: HPuIP.

For semantic segmentation tasks, PointNet++ typically adopts a point-wise cross-entropy loss function, which optimizes the network to predict per-point semantic labels. In this study, point clouds of key fastener components are manually annotated to construct the training dataset. Taking the WJ-8 fastener as an example, the annotation scheme is visualized in the colored point cloud shown in (Fig 8). The trained PointNet++ model enables precise semantic segmentation of fastener components and outputs individual point cloud subsets for each part. These segmented point clouds lay a solid foundation for subsequent geometric parameter extraction and quantitative analysis, supporting accurate dimension measurement and structural condition assessment of critical fastener components.

#### 3.3.2. Geometric parameters calculation of fasteners.

The “ω-shaped” fastener is primarily composed of bolts, metal clips, insulated blocks, and height adjustment pads including Height pad under rail (HPuR) and Height pad under iron pad (HPuIP) as well as other components. Among these components, the bolt presses the metal clip to secure the rail to the sleeper; hence, the height of the bolt’s upper surface reflects the tightness of the fastener. The insulated block is used for the fine adjustment of the rail gauge, while the height adjustment pads are used for adjusting the rail height. These two types of pads are available in different specifications.

This study demonstrates the point cloud-based geometric parameter measurement method using the WJ-8 fastening system as a case study. For each fastener, multiple geometric parameters need to be systematically calculated to assess its structural integrity and adjustment status. The proposed method is generalizable to other fastener systems, including the Vossloh-300 series, WJ-7, and other similar elastic railway fasteners. (Fig 8) presents the structural schematic, in-situ photograph, and point cloud representation of the WJ-8 fastening system.

**Fig 9:**
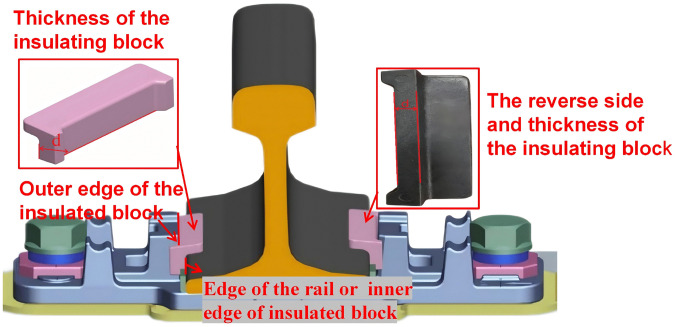
The installation and structure of the insulated blocks.


**(1) The specification of the insulated block.** Insulated blocks are manufactured in five standard specifications (designated as No. 7 through No. 11), with corresponding nominal thicknesses ranging from 7 to 11 mm in 1 mm increments. Track gauge adjustment is achieved by selectively replacing insulated blocks, where the appropriate thickness is chosen to achieve the desired gauge modification. (Fig 9) illustrates the installation configuration and structural details of the insulated block, where d denotes the block thickness. Given that the inner surface of the insulated block interfaces directly with the rail edge, its thickness can be computed based on the difference between the x-coordinates of the insulated block’s outer edge and the adjacent rail edge. The insulated block thickness d is determined as follows:

$$\begin{array}{c} d=absolute(X_{RailEdge}-X_{InsuOutEdge}) \end{array}$$ (2)

Where *X*_*RailEdg*e_ and *X*_*RailEdg*e_ are x-coordinates of the outer edge of the insulated block and adjacent edge of the rail, respectively. To accurately calculate *X*_*RailEdg*e_ and *X*_*RailEdg*e_, this paper fits lines to point clouds of the outer side of the insulated block and the edge of the rail obtained from PointNet++. The x-coordinates of these lines are values of *X*_*RailEdg*e_ and *X*_*RailEdg*e_.

(2) The height adjustment pads. The WJ-8 type fastener comes with two types of height adjustment pads: the HPuR and the HPuIP. The former is installed between the rubber pad and the iron pad, offering four thickness specifications: 1, 2, 5, and 8 mm. The HPuIP is installed beneath the iron plate and above the buffer pad, available in two thickness specifications: 10 and 20 mm. According to the high-speed railway maintenance standards, when the rail elevation adjustment is less than or equal to 10 mm, only the HPuR needs to be installed; the HPuIP shall be applied when the adjustment requirement exceeds 10 mm. A single set of fastener system can accommodate a maximum of two height adjustment pads, with a total thickness not exceeding 10 mm for the HPuR. These two types of height adjustment pads can raise the rail by a maximum of 30 mm.

The 3D imaging system can scan a portion of the point cloud of the upper surface of the rubber pad. Therefore, the thickness of the HPuR can be calculated using the elevations of the rubber pad’s top surface, the iron pad’s top surface, and the rubber pad’s installed thickness.

The thickness of the HPuR can be determined by:


$$\begin{array}{c} T_{R}=H_{Rubber}-H_{IronUpper}-T_{Rubber} \end{array}$$ (3)

Where $H_{Rubber}$ is the height of upper surface of rubber pad, marked as ⑥. $H_{IronUpper}$ is the upper surface of iron plate, marked as ④. $T_{Rubber}$ is the thickness of rubber pad (for the WJ-8 type fastener, this value is 10 mm typically).

The thickness of HPuIP is calculated using Equation (4).


$$\begin{array}{c} T_{P}=H_{IronUper}-H_{Sleeper}-T_{Buffer}-T_{Iron} \end{array}$$ (4)

where parameter $H_{IronUper}$ denotes the vertical dimension of the iron plate limit pillar’s upper surface marked with the red rectangle. $H_{Sleeper}$ represents the height of upper surface of the sleeper support, marked as ⑧. $T_{Buffer}$symbolizes the vertical dimension of the buffer layer positioned underneath the iron plate, $T_{Iron}$ indicates the elevation discrepancy existing between the upper plane of the iron plate’s positioning pillar and the central upper region of the iron plate itself (is 38.2 mm typically for the WJ-8 type fastener).

(3) Bolt height analysis for structural defect detection. Bolt height reflects the tightness state of fasteners, particularly for excessively loose fasteners. The bolt height parameter ($H_{Bolt}$) can be determined as follows.


$$\begin{array}{c} H_{Bolt}=H_{BoltUpper}-H_{IronUpper} \end{array}$$ (5)

where *H*_*BoltUpper*_ denotes the bolt’s upper surface labeled as ①.

The value of $H_{Bolt}$ represents the looseness of the fastener. If $H_{Bolt}$is higher than a threshold value, the fastener is considered loose; conversely, if $H_{Bolt}$ is lower than a threshold value, the fastener is considered over-tight. Therefore, bolt height can be utilized to detect structural defects in fasteners.

## 4. Experimental results and analysis

### 4.1. Evaluation of the measurement accuracy of the 3D imaging system

**Fig 10:**
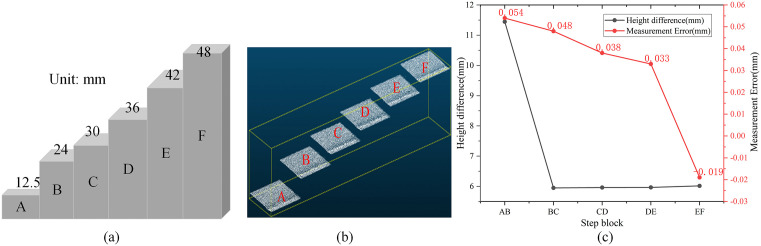
The 3D line laser sensor scans the standard block for analyzing the measurement accuracy of the 3D imaging system. **(a)** Schematic of standard step block; **(b)** Point cloud of step block; **(c)** Measurement error of block.

The precision and reliability of the 3D imaging system are fundamental for accurate defect detection and geometric parameter measurement. To evaluate the accuracy of the 3D imaging system, we used the 3D line laser sensor to scan a standard step block, as shown in (Fig 10(a)). This block has height differences of 6 mm and 11.5 mm, with a manufacturing accuracy of ±0.02 mm. The sensor’s field of view is trapezoidal, as depicted in (Fig 4(b)), with the near end being wider. The measurement accuracy of the sensor increases as the measured object is closer to the wider near end of its field of view. To ensure the standard block was tested under the same conditions as railway track inspection, we mounted the sensor on the track inspection vehicle at the same mounting height and distance. The vehicle was used to scan the step block to generate its point cloud, as illustrated in (Fig 10(b)). We determined the height differences for six different step surfaces and calculated the measurement errors relative to the known ground truth, as shown in (Fig 10(c)). The experimental results show that the measurement errors of the system fall in the range of 0.02 mm to 0.05 mm.

### 4.2. Dataset construction and evaluation metrics

An extensive RGB-P bimodal dataset was constructed for this study to support three core tasks. These tasks cover visual defect detection and fastener localization, point cloud segmentation and high-precision geometric parameter measurement. The dataset was collected from three distinct sites across China. These sites include the Nanchang-Hangzhou mainline railway, the Nanchang-Changsha mainline railway, and a dedicated experimental test track.

#### 4.2.1. Overall dataset characteristics.

The full dataset contains 20000 RGB-P bimodal scans, yielding over 30000 individual fastener instances. It covers both ballasted and ballastless track systems, and includes three mainstream fastener types deployed in Chinese railways. These types are the WJ-2, WJ-7, and WJ-8 systems. A total of 1900 visually defective fastener instances are included. These instances represent common defects found in operational lines.

To ensure comprehensive validation across different scales and imaging conditions, the dataset includes two complementary image formats. Long-strip track images contain multiple consecutive fasteners. These images simulate the continuous scanning mode of on-board inspection systems. Cropped single-fastener images are used for detailed component analysis. All samples exhibit significant variations in illumination, color cast, and texture clarity. They also present typical on-site interferences such as oil contamination and rust. This diversity effectively replicates the complex real-world imaging environments encountered in routine railway track inspections.

#### 4.2.2. Dataset partition for visual defect detection and fastener localization.

To rigorously evaluate the cross-line generalization capability of the YOLOv8 model for visual defect detection and fastener localization, the dataset was divided into training, validation, and test sets according to the data collection line, rather than using random sampling. This partition strategy ensures that the test set contains completely unseen data from a geographically and operationally distinct railway line. It therefore provides a more realistic assessment of model performance in practical deployment.

The training and validation subsets are derived exclusively from data collected on the Nanchang-Hangzhou line and the dedicated experimental test track. The independent test subset is collected solely from the Nanchang-Changsha line. This line is not involved in any stage of model training or validation. The independent test subset contains 4,181 visually normal fastener instances and 196 visually defective fastener instances. It provides a reliable benchmark for evaluating the model’s performance on unseen operational data.

#### 4.2.3. Dataset for Point Cloud Segmentation and Geometric Measurement.

**(1) Point Cloud Segmentation Dataset**. Manual annotation of fastener point clouds is extremely time-consuming and labor-intensive. This process requires precise instance-level segmentation of multiple small, closely spaced components with complex 3D geometries. Therefore, a dedicated point cloud dataset was annotated for training and evaluating the PointNet++ segmentation model. This dataset comprises 580 manually labeled fastener point clouds. All samples are collected from the dedicated experimental test track and cover both WJ-7 and WJ-8 type fasteners.
**(2) Geometric Measurement Accuracy Validation Dataset.** A separate validation dataset was constructed to quantitatively assess the geometric parameter measurement accuracy of the proposed system. This dataset includes 300 intact fasteners. It consists of 150 WJ-7 and 150 WJ-8 type fasteners collected exclusively from the ballastless tracks of the Nanchang-Changsha mainline railways. Manual measurements using vernier calipers and feeler gauges were performed on all these samples. These measurements are used to establish reference ground truth values.

#### 4.2.4. Evaluation metrics.

The performance of the fastener detection model was evaluated using standard object detection metrics including recall, precision, and mean Average Precision (mAP) [35], while the performance of the PointNet++ network was evaluated using the Intersection over Union (IoU) metric [36]. All algorithms were implemented in Python 3.9 (PyTorch 1.12) and executed on an Intel i7-13700KF CPU and an NVIDIA RTX 4070 Ti GPU.

**Fig 11:**
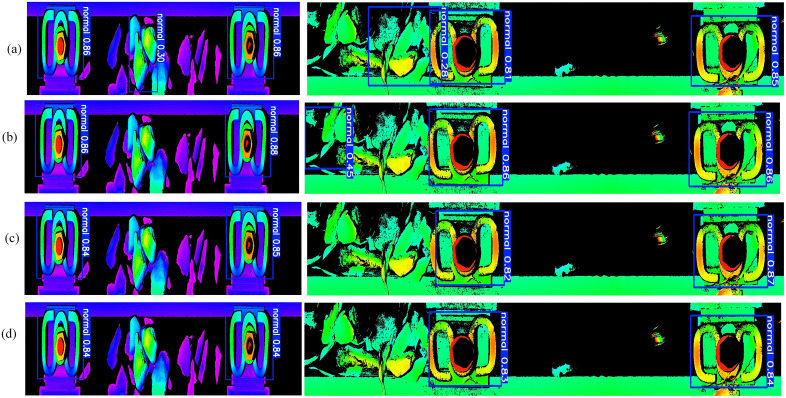
Detection and localization results of visually normal fasteners using different methods. **(a)**-(d) show the detection results of DETR, RT-DETR, YOLOv11s, and YOLOv8s models, respectively.

### 4.3. Visual Defect Detection and fastener location Comparison With State-of-the-Art Networks

**Fig 12:**
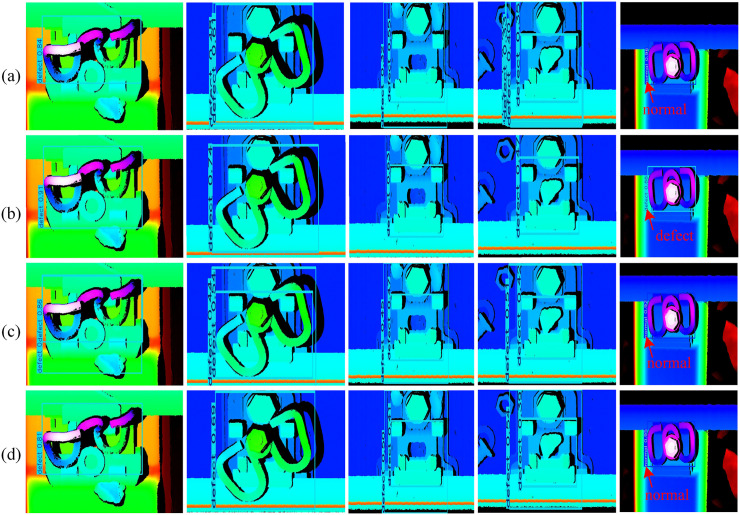
Comparison of visual defect detection results for defective fasteners. **(a)**-**(d)** show the detection results of DETR, RT-DETR, YOLOv11s, and YOLOv8s models, respectively.

To evaluate the performance of the YOLOv8s network, we conducted comprehensive comparisons with three state-of-the-art object detection methods: the YOLOv11s model [37], the Transformer-based end-to-end detector (DETR) [29], and the multi-scale feature fusion CNN-Transformer hybrid architecture (RT-DETR) [28]. All models were trained for 200 epochs using identical training protocols and hardware configurations. The comparative analysis of visual defect detection and fastener location performance across different methods is presented in Table 1 and (Figs 11 and 12).

**Table 1:** Comparison of fastener detection performance across different models.

Models	Parameters	Average Inference Time	Visually Defective Fastener			Visually Normal Fastener		
			Precision	Recall	mAP50	Precision	Recall	mAP50
Yolov8s	11.1M	1.3 ms	97.7%	95.9%	94.8%	99.6%	99.8%	99.6%
Yolov11s	9.4M	1.3 ms	96.9%	95.9%	94.4%	99.4%	99.3%	99%
DETR	41.9M	11.6 ms	100%	92.6%	92.6%	94.8%	99.5%	96.9%
RT-DETR	32.0M	9.0 ms	99.7%	95.9%	95.8%	94.5%	99.7%	97%

Results from Table 1 reveal that DETR achieved 100% precision for visually defective fasteners but exhibited the lowest recall (92.6%) and mAP50 (92.6%) among all models. More critically, the average inference time of DETR is approximately 9 times slower than YOLOv8s. The RT-DETR model achieved the highest mAP50 (95.8%) for visually defective fasteners, but its average inference time is approximately 7 times slower than YOLOv8s. For visually normal samples, both DETR and RT-DETR models frequently misclassified background as fastener components, leading to inaccurate fastener region localization. In contrast, YOLOv8s achieved the highest precision (99.6%), recall (99.8%) and mAP50 (99.6%) for visually normal fasteners, correctly identifying visually normal samples and consequently enabling accurate segmentation of fastener regions. Notably, geometric parameter measurement is only performed on visually normal fasteners, as visually defective fasteners are directly flagged for maintenance. In actual operational railway environments, visually defective fasteners account for less than 0.1% of all fasteners, meaning over 99.9% of system processing time is dedicated to localizing and measuring visually normal fasteners. Combined with the critical requirement of real-time inference for on-board inspection systems, the YOLOv8s model is proven to be the most suitable choice for practical applications.

### 4.4. Performance evaluation for point cloud segmentation using PointNet++

The PointNet++ architecture enables granular control over the output point cloud density, supporting both downsampled and full-resolution semantic segmentation. While downsampling the output point cloud can significantly accelerate inference, we retain the full input point cloud resolution for all segmentation tasks without any downsampling. This design choice is driven by the stringent accuracy requirements for geometric measurements of insulated blocks: higher point cloud density directly enhances the precision of edge localization and thickness quantification, which is indispensable for achieving the sub-millimeter measurement accuracy mandated by high-speed railway maintenance standards.

**Fig 13:**
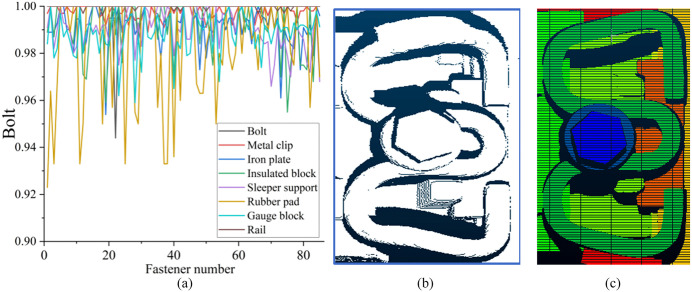
Segmentation results of individual components in the fastener region point cloud. **(a)** IoU values for segmentation of fastener components; **(b)**–**(c)** Point cloud of the fastener region and corresponding segmentation results.

To ensure accurate geometric parameter extraction, we implement full-component segmentation that includes both measurement-critical components and auxiliary elements necessary for system integrity. While a comprehensive functional analysis of all segmented components is beyond the scope of this study, we focus on eight principal categories aligned with the labeling scheme defined in Fig 7. The PointNet++ network was pre-trained on ShapeNet [38]and fine-tuned with 580 annotated point cloud dataset. The model was trained using a random 7:1.5:1.5 train-validation-test split ratio. Representative segmentation results are presented in (Fig 13). Representative segmentation results are presented in (Fig 13). The trained PointNet++ network achieved near-perfect segmentation performance (IoU: 0.99–1.00) for most components, with only minor deviations observed in rubber pad segmentation. This minor deviation is attributed to the fact that most of the rubber pad is occluded by the rail, leaving only a narrow exposed strip, which leads to sparse point cloud coverage. However, since the rubber pad thickness is a standardized nominal value and is not a target measurement parameter in this study, this segmentation error has no impact on the final geometric parameter quantification results. These results confirm that the trained PointNet++ network can accurately segment complex fastener point clouds at full resolution, providing high-fidelity component-wise point clouds for subsequent high-precision geometric parameter quantification.

### 4.5. Verification of geometric parameters measurement accuracy

Measurement error is defined as the difference between the measurements obtained by the proposed method and the corresponding ground truth reference values.

**Fig 14:**
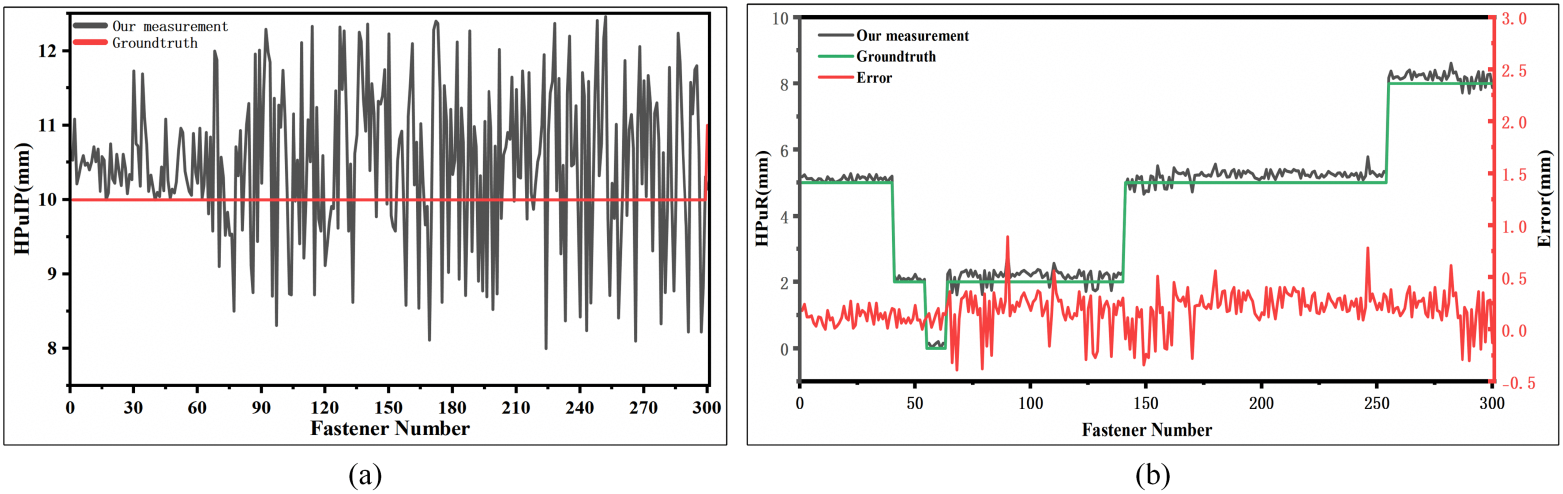
Measurement results for height adjustment pads. **(a)** and **(b)** show HPuIP and HPuR thickness measurements across 300 fasteners, respectively.

**(1) HPuIP Thickness Measurement Results**. HPuIP components are produced in two standard thicknesses: 10 mm and 20 mm, corresponding to a 10 mm specification step. The measurement results are presented in (Fig 14(a)). A small number of measurement errors exceed 2 mm, which can be attributed to local surface irregularities of individual sleeper supports. However, since the thickness difference between adjacent HPuIP specifications is 10 mm, measurement errors within 5 mm do not compromise correct specification identification. These findings demonstrate that the proposed method can reliably determine the thickness specifications of HPuIP despite these minor measurement deviations.
**(2) HPuR Thickness Measurement Results**. HPuR components are manufactured in standard thicknesses of 1 mm, 2 mm, 5 mm, and 8 mm, corresponding to specification steps of either 1 mm or 3 mm. (Fig 14(b)) shows the HPuR thickness calculation results for 300 fasteners using the proposed method. Field verification revealed that the actual HPuR thicknesses in the test section are 0 mm, 2 mm, 5 mm, and 8 mm. A measured value of 0 mm indicates the absence of an HPuR component, which is a typical fastener defect state. The experimental results show that 296 fasteners have measurement errors within 0.5 mm, while the remaining 4 fasteners, which exhibited heavy surface oil contamination, had larger measurement errors ranging from 0.5 mm to 1 mm. By rounding the measured values to the nearest standard thickness, the proposed method can accurately identify the HPuR thickness specifications. The measurement results fully meet the accuracy and reliability requirements of high-speed railway maintenance standards.

**(3) Insulated Block Specification Measurement Results.** The specifications of insulated blocks are determined by measuring the distance from the rail edge to the outer surface of the insulated block. The accuracy of this measurement depends significantly on the horizontal sampling interval dx. A smaller dx value produces a smaller horizontal spacing between adjacent measurement points, resulting in higher resolution point cloud data. The line laser sensor employed in this research supports dx values ranging from 0.1 mm to 0.5 mm. Increasing the dx value increases the sensor’s scanning speed but simultaneously reduces the point cloud density, leading to sparser data representation and lower edge detection accuracy.

**Fig 15:**
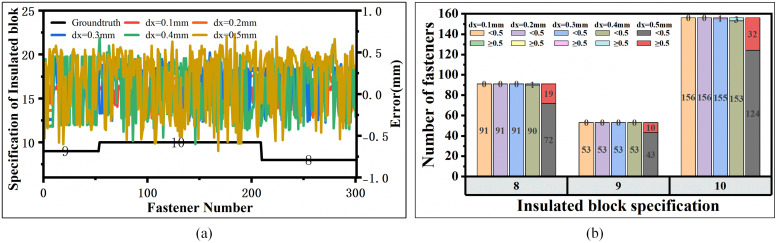
Measurement error analysis for insulated block specifications at varying sampling intervals. **(a)** Measurement error distribution across different dx values. **(b)** Statistical count of fasteners with measurement errors exceeding the 0.5 mm tolerance.

To quantitatively evaluate the impact of different sampling intervals on insulated block measurement accuracy, we conducted systematic experiments using dx values of 0.1 mm, 0.2 mm, 0.3 mm, 0.4 mm, and 0.5 mm. The test set included 300 fasteners with three distinct insulated block specifications: 8 mm, 9 mm, and 10 mm. For analytical clarity, the fasteners were sorted in ascending order of their insulated block specifications. The complete measurement results and corresponding error distributions for each sampling interval are presented in (Fig 15).

Our experimental results demonstrate a clear positive correlation between sampling interval and measurement error: as dx increases, the measurement error increases monotonically. Since insulated block specifications differ by exactly 1 mm increments, a measurement error exceeding 0.5 mm will lead to incorrect specification identification. When dx is set to 0.5 mm, approximately 20% of the measured fasteners exhibit errors greater than the acceptable tolerance threshold of 0.5 mm. In contrast, when dx is reduced to 0.4 mm or less, no fasteners show measurement errors exceeding this critical tolerance limit. In practical engineering applications, the horizontal sampling interval dx should be no greater than 0.4 mm. A value approaching 0.4 mm is recommended to achieve the optimal trade-off between measurement accuracy and computational efficiency.

**(4) Bolt Height Measurement Results.**The 300 test fasteners encompass three distinct categories: over-tight fasteners, normal fasteners, and loose fasteners. Relevant railway track maintenance standards define these categories based on clip gap measurements or direct clamping force testing: fasteners with clip gaps less than 0.5 mm are classified as over-tight, those with gaps exceeding 1 mm are categorized as loose, and fasteners with gaps between 0.5 mm and 1 mm are considered to have normal clamping force [39]. Direct measurement of clamping force is extremely time-consuming, so researchers usually evaluate fastener tightness based on bolt height or clip gap measurements [20,22], and this study selects bolt height measurement as the core indicator for evaluating fastener tightness.

**Fig 16:**
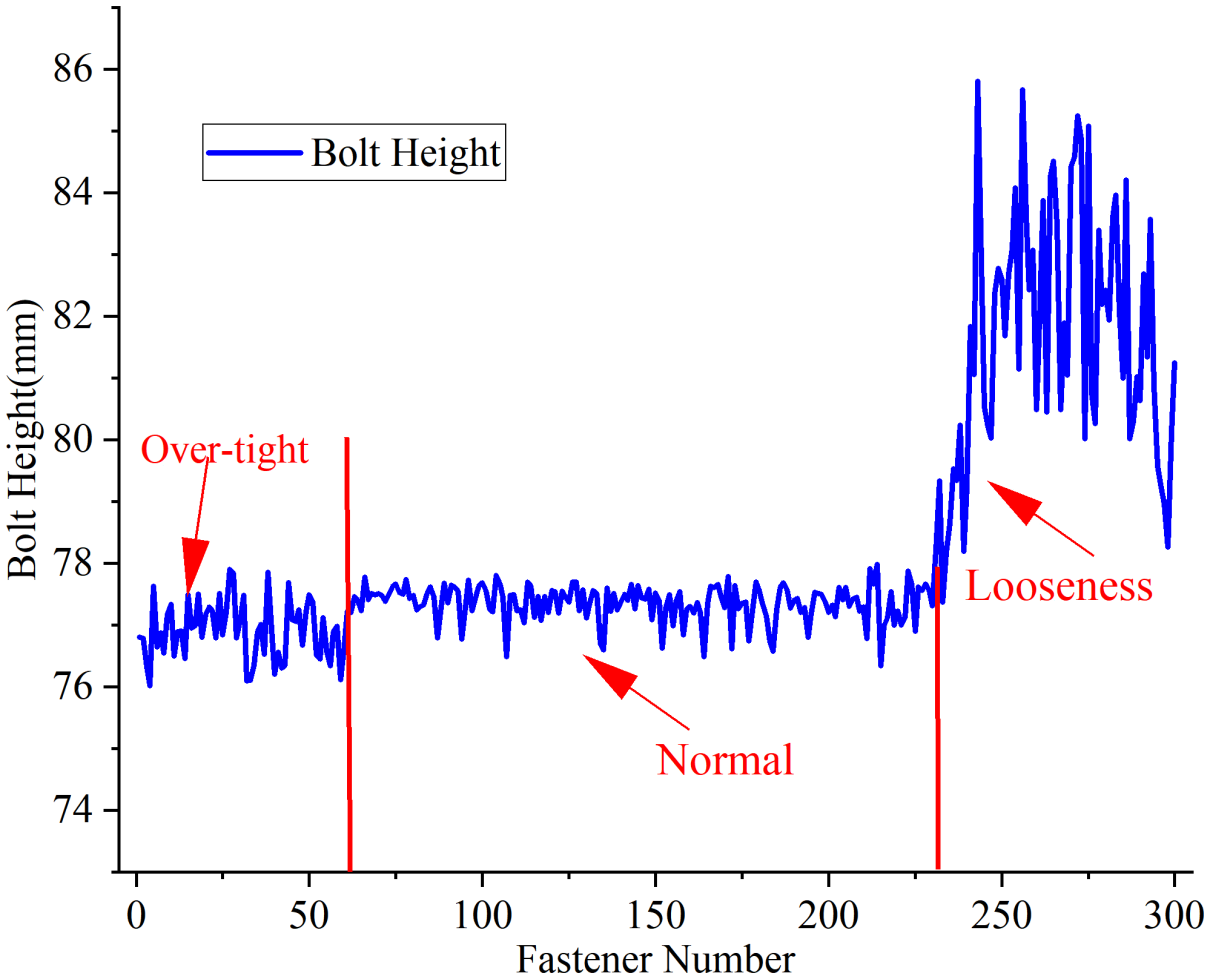
Bolt height measurements for different fastener categories.

The bolt height measurement results for these three fastener categories are presented in (Fig 16). Analysis of the test data reveals that both normal and over-tight fasteners have bolt heights statistically concentrated around 77 mm: normal fasteners typically exhibit bolt heights slightly higher than 77 mm, although some specimens fall below this threshold, while over-tight fasteners generally display bolt heights slightly lower than 77 mm, though certain samples also measure above this value. Manufacturing tolerances and minor surface contaminants on the bolts result in substantial overlap between the bolt height distributions of normal and over-tight fasteners, thereby rendering them nearly indistinguishable through height measurement alone. In contrast, loose fasteners exhibit consistently and substantially higher bolt heights than both normal and over-tight fasteners, creating a clear separation in the measurement data. This distinct height difference enables the proposed method to achieve reliable detection of loose fastener conditions, which is critical for ensuring railway track safety in daily practical operation.

### 4.6. Structural Defects Detection Analysis Based on Bolt Height

Bolt Height Measurement Results demonstrate that bolt height can reliably distinguish over-loose fasteners from normal and over-tight fasteners, this section further optimizes the detection threshold and validates the method on a large-scale dataset.

Since most in-service railway fasteners are in good condition and defective fasteners are promptly repaired during routine maintenance, it is difficult to collect sufficient natural defective fastener data from operational lines. Therefore, we collected 1000 normal fasteners from an operational ballasted railway line, and manually fabricated 230 fastener samples with varying degrees of loosening on a dedicated test track. Specifically, we loosened the bolts to different torque values using an electric torque wrench without altering any other components of the fasteners, resulting in both slightly loose and severely loose samples. An inspection vehicle equipped with a 3D line laser sensor scanned these validated fasteners to acquire RGB-P bimodal data. The final dataset covers both WJ-7 and WJ-8 type fasteners. We calculated the bolt height for each fastener and evaluated the defect identification performance of the proposed method under different threshold settings.

The key to detecting over-loose fasteners via bolt height lies in setting a reasonable threshold: a fastener is classified as over-loose when its bolt height exceeds this threshold. As the bolt heights of normal and over-tightened fasteners are statistically indistinguishable, we used the bolt height distribution of normal fasteners as the reference for threshold calculation. Assuming the bolt heights of normal fasteners follow a normal distribution,we computed the average and variance of the bolt heights of the 1000 normal fastener samples. The calculated average(*avg*) bolt height is 77.27 mm and the corresponding variance (*var*) is 0.2 mm. The detection threshold is defined as:


$$\begin{array}{c} Threshold=avg+a\times var \end{array}$$ (6)

where *a* is an adjustable parameter that controls the trade-off between false positives and false negatives.

**Fig 17:**
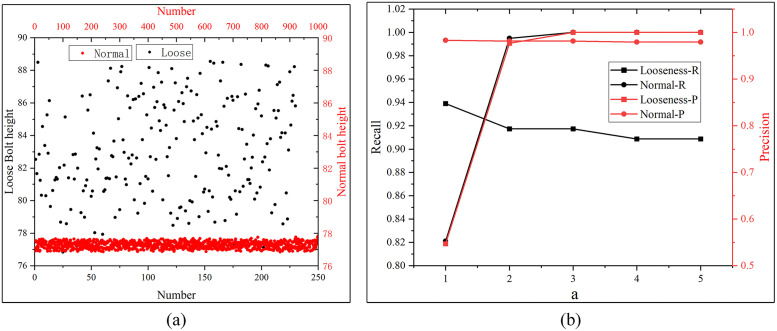
Over-loose fastener detection results based on bolt height. **(a)** Bolt height distribution of normal and over-loose fasteners; **(b)** Precision and recall curves under different threshold parameters.

The bolt height distributions of the 1000 normal fasteners and 230 over-loose fasteners are shown in (Fig 17(a)). There is partial overlap in bolt height between normal and slightly loose fasteners, while severely over-loose fasteners exhibit significantly higher bolt heights that are clearly distinguishable. The precision and recall values for over-loose fastener detection under different values of a are presented in (Fig 17(b)). The experimental results show that when a≥2, the precision and recall for over-loose fastener detection are no less than 91% and 90%, respectively, while the precision and recall for normal fastener identification are no less than 98% and 99%. In practical applications, a higher threshold reduces false positives but increases false negatives (missing slightly loose fasteners), while a lower threshold reduces false negatives but increases false positives (misclassifying normal fasteners as loose). Given that severely over-loose fasteners pose the greatest safety risk to railway operations, bolt height measurement provides an effective and efficient method for their rapid screening in large-scale railway inspections.

It should be noted that the above average and variance values are derived from fasteners on a single test railway line. Absolute bolt height values may vary across different lines, rail ages and environmental conditions due to discrepancies in track construction standards, component manufacturing batches and long-term track settlement. Consequently, the fixed threshold calculated from this specific dataset can only serve as a reference for other lines, and a universal fixed threshold lacks generalizability. We therefore recommend an adaptive threshold approach. During the initial inspection of a new railway line, the system measures the bolt heights of all fasteners in a continuous intact section, derives the normal bolt height distribution by excluding obvious outliers, calculates the line-specific average and variance to set a customized threshold, and flags fasteners exceeding this threshold for on-site verification.

### 4.7. Time analysis for fastener detection and geometric parameter measurement

Time performance analysis was conducted for the entire inspection pipeline, including fastener detection and geometric parameter measurement, based on 100 fastener samples. The data acquisition system was configured with a horizontal sampling interval (*dx*) of 0.4 mm, a maximum sensor line frequency of 1200 Hz, and a vertical trigger interval (*dy*) of 1.0 mm. Under this configuration, the RGB-P bimodal data acquisition speed is 1.2 m/s (equivalent to 4.32 km/h). It is worth noting that increasing the vertical trigger interval reduces point cloud density but increases the data acquisition speed and improves overall efficiency. The spatial dimensions of each fastener region are approximately 90 mm × 200 mm.

The total processing time for 100 fasteners was 31.12 s, corresponding to an average processing time of 0.311 s per fastener. PointNet++ point cloud segmentation is the most time-consuming module, accounting for 0.31 s per fastener. In contrast, fastener localization and visual defect detection based on the proposed YOLOv8 model only take 1.2 ms per fastener. This result indicates that point cloud-based 3D segmentation algorithms have significantly higher computational complexity than 2D image-based object detection algorithms.

Given an average inter-fastener spacing of 0.63 m, processing 100 fasteners corresponds to a total inspection distance of 63 m, resulting in an average system processing speed exceeding 2 m/s. This processing speed is higher than the data acquisition speed (1.2 m/s), which guarantees real-time defect detection and geometric parameter measurement when the system is deployed on hand-pushed track inspection vehicles operating at conventional human walking speeds. For quantitative comparison, manual measurement of 100 fasteners by trained personnel typically requires approximately 60 minutes of continuous work.

## 5. Discussion, limitations and future work

This section discusses the key findings, inherent limitations of the proposed fastener inspection and geometric parameter measurement method, and feasible future research directions to provide a reference for its optimization and engineering application.

As verified obove, the YOLOv8 model achieves the best comprehensive performance for the proposed fastener inspection task by delivering superior localization accuracy for intact fasteners with an mAP50 of over 98%. This high localization accuracy is a prerequisite for point cloud segmentation and geometric parameter measurement. Although DETR and RT-DETR exhibit marginally higher visual defect detection accuracy, their high false positive rate exceeding 5% in normal fastener localization leads to inaccurate region cropping and geometric measurement failures, which justifies the selection of YOLOv8 as the core localization and defect detection model.

The proposed method enables accurate measurement of key fastener geometric parameters, but its performance depends on unobstructed observation of fastener components. It is well-suited for ballastless tracks commonly used in Chinese high-speed railways, where fasteners are rarely covered by ballast or debris. A key limitation is potential measurement errors on ballasted tracks with severe ballast coverage, resulting from incomplete fastener point cloud extraction.

Lubricating oil contamination on bolts, a common occurrence in routine maintenance, and inherent manufacturing tolerances cause significant overlap in bolt height distributions between normal and over-tightened fasteners. Thus, bolt height only reliably indicates severely over-loose fasteners, but cannot distinguish normal from over-tightened conditions. This finding is consistent with previous studies [22,40] and further verifies the nonlinear correlation between clamping force and bolt height reported in previous studies.

Future research will focus on the following four aspects. First, it will improve visual defect detection accuracy and intact fastener localization precision. Second, it will integrate visual measurement with vibration and acoustic features to enhance fastener clamping force detection and distinguish normal from over-tightened fasteners. Third, it will develop point cloud semantic segmentation-based ballast removal algorithms to improve system robustness under ballast coverage for ballasted tracks. In addition, the proposed framework has strong generalization and can be readily extended to other mainstream ω-shaped fasteners such as SFC and W300-1 types.

## 6. Conclusion

This paper presents an integrated method for railway fastener defect detection and geometric parameter measurement based on a 3D line laser sensor. By constructing RGB-P bimodal data with a precise one-to-one mapping relationship, the system leverages the complementary strengths of image-based fast defect detection and point cloud-based high-precision measurement. It realizes simultaneous defect identification and quantitative measurement of key geometric parameters, including insulated block specifications as well as HPuR and HPuIP layer thicknesses.

Extensive field experiments validated the reliability and effectiveness of the proposed method. The YOLOv8 model achieves 97.7% precision and 95.9% recall for visual defect detection, and exhibits superior intact fastener localization accuracy with 99.6% precision and 99.8% recall, both of which are critical for subsequent geometric measurement. All critical geometric parameter measurements satisfy their respective engineering tolerances for railway maintenance. For HPuR components with 1 mm and 3 mm specification steps, 98.7% of measurements have errors below 0.5 mm. HPuIP components with a 10 mm specification step show a maximum measurement error of less than 2.5 mm, well below the 5 mm specification identification tolerance. For insulated blocks with a 1 mm specification step, 99% of measurements have errors below 0.5 mm at horizontal sampling intervals no greater than 0.4 mm. Bolt height measurement delivers no less than 90% precision and 91% recall for severe fastener loosening detection using line-adaptive thresholds. The system achieves a real-time operating speed of 4.32 km/h, which effectively supports on-site inspection at human walking speed.

The detection results and geometric data can be directly applied to railway intelligent operation and maintenance systems. Our method significantly improves the efficiency of fastener inspection and parameter measurement compared with traditional manual inspection and measurement methods. This work provides a practical automated solution for railway fastener inspection and facilitates the intelligent development of track maintenance.
